# Antidepressant-Like Effects of *Cistanche tubulosa* Extract on Chronic Unpredictable Stress Rats Through Restoration of Gut Microbiota Homeostasis

**DOI:** 10.3389/fphar.2018.00967

**Published:** 2018-08-21

**Authors:** Yang Li, Ying Peng, Ping Ma, Hanlin Yang, Haiyan Xiong, Mengyue Wang, Chongsheng Peng, Pengfei Tu, Xiaobo Li

**Affiliations:** ^1^School of Pharmacy, Shanghai Jiao Tong University, Shanghai, China; ^2^State Key Laboratory of Natural and Biomimetic Drugs, School of Pharmaceutical Sciences, Peking University, Beijing, China

**Keywords:** *Cistanche tubulosa*, antidepressant, chronic unpredictable stress, gut microbiota, microbiota–gut–brain axis

## Abstract

Growing evidence shows that neuropsychiatric disorders, such as depression, are linked with gut microbiome through the gut–brain axis. Cistanches Herba is well known for the treatment of “kidney-yang” deficiency in traditional Chinese medicine (TCM), and has been used for treatment of neurodegenerative diseases in recent years. In this study, chronic unpredictable stress (CUS)-induced depression model was established to explore the impact of *Cistanche tubulosa* extract (CTE) on behavioral tests, monoamine neurotransmitters and neurotrophic factors in hippocampus and colon, gut microbiota composition, and short-chain fatty acids (SCFAs) production. Moreover, correlation analysis was used to evaluate the functional relationship between altered gut microbiota, changed neurotransmitters and neurotrophins in hippocampus and colon, and disturbed concentration of SCFAs. CTE significantly improved depression-like behaviors in rats under CUS. Brain level of 5-hydroxytryptamine (5-HT) and brain-derived neurotrophic factor (BDNF) expression in CUS rats were restored by CTE. The relative abundance of gut microbiota and the concentrations of acetate and hexanoic acid could also be modulated by CTE treatment. We further showed that the application of CTE in CUS rats led to strong correlation among disrupted gut microbiota composition, hippocampus neurotransmitter levels, and production of neuroactive metabolite SCFAs. Altogether, these results identify CTE as a potential treatment for depressive symptoms by restoring homeostasis of gut microbiota for microbiota–gut–brain axis disorders, opening new avenues in the field of neuropsychopharmacology.

## Introduction

Cistanches Herba (Rou Cong-Rong in Chinese) is officially recorded as the dried succulent stems of *Cistanche deserticola* (Y. C. Ma) and *Cistanche tubulosa* (Schrenk) in Chinese Pharmacopoeia Commission (2015). It is a well-known traditional Chinese medicine (TCM) for treating kidney deficiency, impotence, female infertility, morbid leucorrhea, profuse metrorrhagia, and senile constipation (Chinese Pharmacopoeia Commission, 2015). Previous studies have revealed several main chemical constituents of Cistanches Herba, including phenylethanoid glycosides (PhGs), iridoids and iridoid glycosides, lignans, alditols, oligosaccharides, and polysaccharides. Pharmacological analysis has shown that Cistanches Herba exhibits a broad range of neuroprotective, immunomodulatory, anti-inflammatory, and hepatoprotective activities (Jiang and Tu, 2009; Fu et al., 2017). PhGs have been regarded as the major active components of Cistanches Herba possessing various pharmacological activities, such as neuroprotective, immunomodulatory, anti-inflammatory, hepatoprotective, and anti-oxidative, etc. (Jiang and Tu, 2009; Fu et al., 2017). Cistanches Herba was originally recorded in the Classic of Herbal Medicine (“Shennong Bencao Jing” in Chinese), a well-known book on medicinal plants written between about 200 and 250 AD, as a “high herb” that can regulate “five (viscera) strains and seven (overeating, anger, moisture, cold, worry, wind and rain, and fear) impairments” (Huang, 1982). Nowadays Cistanches Herba is widely accepted as a herbal tonic for general debility (Fu et al., 2017). *C. tubulosa* glycoside capsules (Memoregain^®^) are in use for the treatment of Alzheimer’s disease (Guo et al., 2013). A dysfunctional dopaminergic system is one of the key pathogenesis of monoamine hypothesis of depression (Duman et al., 1997; Krishnan and Nestler, 2008). Echinacoside (one of the PhGs) and PhGs from *Cistanche salsa* have been found to protect dopaminergic neurons against dopamine (DA) neurotoxicity induced by 1-methyl-4-phenyl-1,2,3,6-tetrahydropyridine (MPTP) which can increase DA levels in the striatum of C57 mice (Tian and Pu, 2005; Geng et al., 2007). Additionally, catalpol and geniposide are two representative iridoids in Cistanches Herba, and they have been found to ameliorate chronic unpredictable stress (CUS)-induced depression-like behavior via restoring hypothalamic–pituitary–adrenal (HPA) axis dysfunctions and catalpol can upregulate brain-derived neurotrophic factor (BDNF) expression (Cai et al., 2015; Wang et al., 2015). Moreover, the latest publication gives a fresh evidence that Cistanches Herba decoction significantly reduced the immobility period in the mouse tail suspension test, which strongly suggesting Cistanches Herba possesses potential antidepressant-like qualities (Wang D. et al., 2017).

Traditional Chinese medicine attracts increasing attention as a treatment of depression because of its comparatively moderate side effects (Sarris et al., 2011; Feng et al., 2016). Various types of TCMs, such as individual compounds from crude drug (ginsenoside Rb1, ginsenoside Rg3, and Yuanzhi-1) (Jin et al., 2015; Kang et al., 2017; Wang G. L. et al., 2017), single Chinese medicinal materials (*Panax ginseng* root, *Acorus tatarinowii* rhizome, and *Morinda officinalis*) (Dang et al., 2009; Han et al., 2013; Xu et al., 2016), and TCM decoctions (Kai-Xin-San, Chaihu-Shu-Gan-San, Xiaoyaosan, and Xiaochaihutang) (Dai et al., 2010; Su et al., 2011; Su et al., 2014; Yan et al., 2016) have been deeply studied and shown to reverse or mitigate depression-like symptoms. For example, Kai-Xin-San was found to markedly alleviate symptoms of CUS-induced depressive rats by increasing amount of neurotransmitter, neurotrophic factors, and their corresponding receptors, and promoting the expression of synaptotagmin and dendritic spine density (Yan et al., 2016).

Nevertheless, previous studies of TCM rely on results in depressant-like behavioral tests and focus on the conventional pharmacological mechanism of action. The connection between the antidepressant efficacy of orally administered TCM and gut microbiota is not well understood. Growing evidence supports that gut microbiota plays a crucial role in regulating brain functions, particularly depression and other stress-related disorders (Foster and Neufeld, 2013; Sherwin et al., 2017). For instance, chronic stress results in dysregulation of the rat gut microbial structure, with a decrease in Firmicutes/Bacteroidetes ratio, and more specifically, decrease in the relative abundance of *Lactobacillus* and increase in *Oscillibacter* (Meng et al., 2017). Furthermore, fecal transplantation of dysregulated microbiota from depressed patients to germ-free mice conferred a depression-like phenotype in these animals, indicating that dysregulated gut microbiota causes behavioral and physiological symptoms of depression and anxiety (Kelly et al., 2016; Zheng et al., 2016). In animal experiments, some microbiota modulators such as probiotics and prebiotics were found to improve depression-like behavior in chronically stressed mice by ameliorating the gut microbiota (Bravo et al., 2011; Bharwani et al., 2017; Burokas et al., 2017).

Given the association between gut microbiota and disease development, as well as the plasticity of gut microbiota structure, we decided to investigate the effects of in gut microbiota for mediating depression attracted our attention and was investigated (Chang et al., 2017; Xu et al., 2017). To achieve this, 16S rRNA gene sequencing combined Metastats analysis (White et al., 2009) was performed to analyze gut microbiota composition. In the present study, *C. tubulosa* was selected to evaluate its antidepressant-like effects because it has a higher level of PhGs compared to *C. deserticola*. A rat CUS-induced depression model was established to explore the impact of *C. tubulosa* extract (CTE, consisted of 48.6% PhGs, 6.9% iridoid glycosides, and 20.0% total saccharides) on behavioral tests, monoamine neurotransmitters and neurotrophic factors in hippocampus and colon, gut microbiota composition, and short-chain fatty acid (SCFA) production. Moreover, the functional relationship between altered gut microbiota, changed neurotransmitters and neurotrophins in both hippocampus and colon, and disturbed concentration of SCFAs was studied. Correlation analysis was used to substantiate evidence that CTE targets gut microbiota for the treatment of depression by modulating the microbiota–gut–brain axis.

## Materials and Methods

### Materials

Fluoxetine (FLX) was purchased from Aladdin Industrial Inc. (Shanghai, China). 5-hydroxytryptamine (5-HT), norepinephrine (NE), and BDNF ELISA kits were purchased from Nanjing Jiancheng Bioengineering Institute (Nanjing, China). HPLC-grade acetonitrile was purchased from Merck (Darmstadt, Germany). Deionized water was prepared from distilled water using a Milli-Q water purification system (Millipore, Bedford, MA, United States). All other reagents and chemicals used were of analytical grade.

Dried stems of *C. tubulosa* were collected from Hetian County (Xinjiang, China). The voucher specimen samples were authenticated by Prof. Xiaobo Li and deposited at the herbarium of the School of Pharmacy, Shanghai Jiao Tong University (Shanghai, China). The homogeneous powdered *C. tubulosa* stems were suspended in tenfold of 70% ethanol, and heat-refluxed three times, each for 1 h at 100°C. The extracts were filtered using gauze, evaporated under vacuum at 65°C and lyophilized. The sample was re-dissolved, and then separated by macroporous resin D101. After adsorption, the fraction eluted with water was discarded. CTE was obtained after eluted with 40% ethanol, then evaporated under vacuum at 65°C and lyophilized.

### Analysis of Chemical Composition of CTE

Relative content of PhGs in CTE was determined by UV spectrophotometry at 330 nm using echinacoside as the standard. The relative content of iridoids and iridoid glycosides was measured by UV spectrophotometry with dual-wavelength method to avoid the spectral interference from PhGs (237 nm was used for spectroscopic quantification, an isosbestic point 280 nm was used for reference wavelength), geniposide was used as the standard. The total carbohydrate content of CTE was determined by phenol-sulfuric acid colorimetric method with glucose as the standard (Chow and Landhäusser, 2004).

Main chemical constituents of CTE were characterized by UPLC-Q-TOF-MS. UPLC-Q-TOF-MS analysis was performed on a Waters ACQUITY UPLC system (Waters Corp., Milford, MA, United States) with an ACQUITY UPLC BEH C18 column (100 mm × 2.1 mm i.d., 1.7 μm, Waters Corp., United States) by gradient elution using 0.1% formic acid acetonitrile (A) and 0.1% formic acid in water (B) at a flow rate of 0.4 ml/min. The gradient profile was: 0–5 min (A: 5–20%), 5–7.5 min (A: 20–30%), 7.5–10 min (A: 30–70%), 10–11 min (A: 70–100%), and held for 1.5 min. The gradient was recycled back to 5% in 0.5 min, and held for 2.5 min for the next run. The injection volume was 3 μl. The temperature of the column oven was set to 35°C.

Mass spectrometry was carried out using a Waters Vion IMS mass spectrometer (Waters Corp., Milford, MA, United States). Ionization was performed in the negative electrospray (ESI) mode. The MS parameters were as follows: capillary voltage, 2.0 kV; cone voltage, 20 V; source temperature, 120°C; desolvation temperature, 500°C; gas flows of cone and desolvation, 50 and 1,000 L/h, respectively. For accurate mass measurement, leucine-enkephalin was used as the lock mass to generate an [M–H]^-^ ion (*m/z* 554.2615). An MS^E^ (Mass Spectrometry^ElevatedEnergy^) experiment in two scan functions was carried out as follows: function 1 (low energy): *m/z* 50–1,000, 0.25 s scan time, 0.02 s inter-scan delay, 6 eV collision energy; function 2 (high energy): *m/z* 50–1,000, 0.25 s scan time, 0.02 s inter-scan delay, collision energy ramp of 20–45 eV. The data were processed using UNIFI 1.8.1 software (Waters Corp., Milford, MA, United States).

### Animal Experiments

Male Sprague-Dawley rats (200 ± 20 g) were procured from Beijing Vital River Laboratory Animal Technology Company (Beijing, China), and housed in the Laboratory Animal Center of Shanghai Jiao Tong University (Shanghai, China). The animals were housed in groups under controlled room temperature (25 ± 2°C, 55 ± 10% relative humidity) with a 12:12 h light–dark cycle. The rats were allowed free access to regular laboratory rats chow and water for 1 week. The animal facilities and protocols were approved by the Animal Ethics Committee of Shanghai Jiao Tong University (Shanghai, China).

Following 1 week acclimation, 40 naive rats were subjected to a period of 72 h sucrose training and sucrose baseline testing. Rats were divided into five groups (*n* = 8) based on their sucrose preference in the sucrose baseline test (**Supplementary Figure S1**). The control group and CUS group were given saline (10 ml/kg). For the other three groups, CTE at high dose (CTEH) (400 mg/kg), low dose (CTEL) (200 mg/kg), and FLX (10 mg/kg) were intragastrically administered 1 h before the CUS procedure (8:00 a.m. to 9:00 a.m.) over the course of 4 weeks.

Chronic unpredictable stress was developed as described previously (Willner, 1997; Xue et al., 2013), except for the control non-stressed group, a series of stressors were applied to the rats: overnight low-intensity stroboscopic illumination (120 flashes/min), white noise (100 dB) for 1 h, water deprivation for 24 h, empty water bottles for 1 h (after water deprivation), food deprivation for 24 h, forced swimming (5 min), physical restraint (1–2 h), soiled cage for 24 h (200 ml water in 100 g sawdust bedding), tail pinch (1 min), 45° cage tilt for 24 h, shock for 30 min, and overnight illumination (12 h). Stressors were applied continuously and randomly for 4 weeks, details of which are described in **Supplementary Table S1**. Food and water were freely available to the control non-stressed rats, which remained undisturbed in another room, except for the 14 h period of deprivation prior to each sucrose test. One rat died during the CUS procedure. Forced swimming test (FST) was performed after 4 days acute administration and after 28 days of stress, sucrose preference test (SPT) (day 29), open-field test (OFT) (day 31), and novelty-suppressed feeding test (NSFT) (day 33) were performed. The outline of design for CUS and behavioral tests is shown in **Supplementary Figure S2**.

Forced swimming test was performed as described previously (Porsolt et al., 1978). The procedure consisted of pretest and test sessions, using the same apparatus and conditions (diameter 20 cm, height 45 cm, containing 25 cm of water maintained at 26°C). During the pretest session, rats were forced to swim for 15 min; 24 h later, rats were placed in the same apparatus for 5 min swim test session. The swimming behavior within 5 min was observed with a video camera, and the duration of immobility were measured and analyzed.

For SPT, rats were first trained to consume 1% (v/v) sucrose solution for 72 h before the SPT. The sucrose baseline test before CUS procedure and the SPT at the end of CUS were performed as described previously (Xue et al., 2013).

Open-field test was carried out at the end of CUS procedure, the apparatus and method were the same as previously detailed (Xue et al., 2013). A video-tracking system (Shanghai Mobile Datum Information Technology Co., Ltd.) was used to record the total distance traveled.

Novelty-suppressed feeding test was assessed as previously described (Xue et al., 2013) and latency to begin eating within 5 min was recorded and analyzed.

### Measurement of Neurotransmitters and Neurotrophic Factors

After behavioral tests, at least four fecal pellets were obtained from each rat, placed in sterile conical tubes and immediately frozen at -80°C for microbial community and SCFAs analysis. After that, rats were sacrificed; the hippocampus and colon were isolated and weighed immediately. Tissue samples were washed with saline, snap frozen in liquid nitrogen, and kept in -80°C for storage. After sequential thawing at -20°C and 4°C, the tissue samples were homogenized in saline and centrifuged for 25 min at 2,500 rpm/4°C. The resulting supernatant was collected and stored at 4°C until use. The levels of 5-HT in hippocampus and colon, NE in hippocampus, and BDNF in hippocampus were determined by commercial ELISA kits according to the manufacturer’s protocols (Hou et al., 2017).

### Compositional Profile Analysis of Gut Microbiota

The total DNA of gut microbiota from fecal samples was extracted as previously described (Wei et al., 2004). Each fecal sample (0.2 g) was blended in 1 ml PBS, and fully homogenized twice, then centrifugation at 200 g for 6 min. The supernatant was added with 20 μl 20% PVP and centrifuged at 300 *g* for 6 min. The resulting supernatant was then centrifuged at 12,000 *g* for 6 min and cell pellets were harvested. Cell pellets were washed by PBS and then were re-suspended in 300 μl lysate I (150 mM NaCl, 100 mM EDTA⋅Na_2_⋅2H_2_O, pH 8.0). The suspension was mixed with 100 μl lysozyme solution (100 mg/ml) and 20 μl 1% RNase, 37°C warm-bathed for 30 min, then added 300 μl lysate II (100 mM NaCl, 500 mM Tris base, pH 8.0). The cell suspension was gently mixed with 50 μl 20% SDS, ice-bathed for 5 min. DNA was then purified by sequential extraction with 1% PVP, Tris-equilibrated phenol and chloroform-isoamyl alcohol (vol/vol/vol/vol, 3.125:25:24:1), and chloroform-isoamyl alcohol (vol/vol, 24:1) followed by precipitation at -20°C for 2 h with two volumes of ethanol and 50 μl 3 M NaAc. DNA was collected by centrifugation, air-dried and dissolved in 100 μl sterile deionized water. The nucleic acid purity was tested using a NanoDrop 2000c Spectrophotometer (Thermo Fisher Scientific, Waltham, MA, United States). Total genomic DNA was then subjected to PCR amplification using primers specific to the V3–V4 regions of the 16S rRNA gene: forward primer 338F (5′-ACTCCTACGGGAGGCAGCA-3′) and the reverse primer 806R (5′-GGACTACHVGGGTWTCTAAT-3′). PCR amplicons were purified with Agencourt AMPure Beads (Beckman Coulter, Indianapolis, IN, United States) and quantified using the PicoGreen dsDNA Assay Kit (Invitrogen, Carlsbad, CA, United States). After the individual quantification step, amplicons were pooled in equal amounts, and pair-end 2 × 300 bp sequencing was performed using the Illumina MiSeq platform with MiSeq Reagent Kit v3 at Shanghai Personal Biotechnology Co., Ltd (Shanghai, China). Paired-end reads were assembled using FLASH software (version 1.2.7), raw sequence reads were quality trimmed using the QIIME software (version 1.8.0) to remove mismatched barcodes and sequences below length thresholds. Afterward, the filtered sequences were clustered into operational taxonomic units (OTUs) with a threshold of 97% sequence similarity using QIIME with UCLUST. Beta diversity was performed on QIIME for weighted UniFrac principal coordinate analysis (PCoA). Finally, Metastats software was used to compare the differences in gut microbial taxonomic composition among different groups.

### SCFAs Concentration Analysis

The concentration of SCFAs (including acetate, propionate, butyrate, isobutyrate, valeric acid, isovaleric acid, and hexanoic acid) in rat fecal samples were determined by GC-2010 gas chromatograph (Shimadzu, Japan), fitted with a DB-FFAP column (30 m × 0.25 mm × 0.25 μm, Agilent, United States). Standard solutions of acetate, propionate, butyrate, isobutyrate, valeric acid, isovaleric acid, and hexanoic acid were prepared at 1, 0.4, 0.2, 0.1, 0.05, 0.01, and 0.005 mg/ml with ether (including 2-methylvaleric acid 0.1 mg/ml as internal standard), respectively. Each fecal sample (0.2 g) was blended in 0.8 ml deionized water by vortexing and centrifuging at 5,000 rpm for 20 min at 4°C, 450 μl of supernatant was added by 50 μl 50% H_2_SO_4_ and 500 μl internal standard solution, vortexing for 1 min and centrifuged at 12,000 rpm for 10 min, then the mixture was placed in 4°C for 30 min. The supernatant was taken for GC analysis. The analysis of SCFAs was carried out as previously described (Wang et al., 2016).

### Statistical Analysis

All of the data were presented as means ± SEM. Statistical analysis was performed using the SPSS 21.0 software (SPSS Inc., Chicago, IL, United States) with a one-way ANOVA followed by Dunnett’s test or Student’s *t*-test. Data were excluded from analyses if greater than two standard deviations from the mean. Values of *p* < 0.05 were considered statistically significant. Pearson’s correlation analysis was employed to evaluate the association between the relative abundance of gut microbiota, SCFAs, hippocampus 5-HT, NE, and BDNF levels, and colon 5-HT.

## Results

### Analysis of Chemical Composition of CTE

Chemical composition of CTE was first comprehensively analyzed. As shown in **Table 1**, PhGs were the main component of CTE, with a relative content of 48.6%, iridoids and iridoid glycosides were 6.9%, and total saccharides were 20.0%. These components of CTE were then characterized using UPLC-Q-TOF-MS. Total of 27 constituents were detected and identified, including 20 constituents of PhGs, five of iridoids and iridoid glycosides, and two of oligosaccharides. Detailed information including retention time, accurate MS, and MS/MS fragment ions are listed in Supporting Information (**Supplementary Table S2**). UPLC-Q-TOF-MS total ion chromatogram (TIC) of CTE is shown in **Supplementary Figure S3**.

**Table 1:** chemical composition of *C. tubulosa* extract.

Compound category	Percentage of relative content	Constituents
PhGs	48.6%	Decaffeoylacteoside
		Cistantubuloside C_1_/C_2_
		Cistanoside H
		Campneoside II
		Echinacoside
		Isomer of campneoside II
		Poliumoside
		Isopoliumoside
		Cistanoside A
		Tubuloside A
		Acteoside
		Isoacteoside
		Cistanoside C
		2′-Acetylacteoside
		Osmanthuside B or osmanthuside B6
		Isocistanoside C
		Tubuloside B
		Osmanthuside B or osmanthuside B6
		Salsaside F or isomer
		Salsaside F or isomer
Iridoids and Iridoid glycosides	6.9%	8-Epiloganic acid or isomer
		8-Epiloganic acid or isomer
		Kankanoside A or isomer
		Kankanoside A or isomer
		Kankanoside N
Glycosides	Not detect	Kankanose
		Cistanoside F
Saccharides	20.0%	–

### CTE Improved the Depressive Behaviors in CUS Rats

In CUS rats, total immobility time in the FST and latency to eat in NSFT were significantly decreased by CTE treatment, and sucrose preference in SPT and total distance covered in OFT were increased.

**FIGURE 1:**
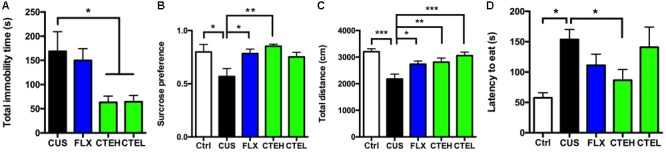
Effects of CTE on behavioral test of CUS rats: **(A)** forced swimming test, **(B)** sucrose preference test, **(C)** open-field test, and **(D)** novelty-suppressed feeding test in CUS rats. Ctrl, control; CUS, chronic unpredictable stress; FLX, fluoxetine; CTEH, *C. tubulosa* extract high dose; CTEL, *C. tubulosa* extract low dose. ^∗^*P* < 0.05, ^∗∗^*P* < 0.01, ^∗∗∗^*P* < 0.001 (*n* = 8, mean ± SEM).

**Figure 1A** illustrates the acute effects of CTE on immobility time in FST in CUS rats. Compared with CUS model group, 3-day acute oral administration of CTE significantly reduced the total immobility time in FST [one-way ANOVA, *F*(3,26) = 4.983, *p* = 0.007]. Further *post hoc* analysis revealed that administration of CTE with high dose group (Dunnett’s test, *p* < 0.05) and low dose group (Dunnett’s test, *p* < 0.05) showed a marked reduction on the total immobility time in the FST.

The effects of CTE on sucrose preference in SPT in CUS rats are shown in **Figure 1B**. Twenty-eight days stress procedure caused a significant decrease in sucrose preference in the CUS model group compared to the control non-stressed rats [one-way ANOVA, *F*(4,34) = 3.993, *p* = 0.009; Dunnett’s test, *p* < 0.05], indicating that the CUS model was successfully developed. As the positive control, FLX group significantly increased the sucrose preference in CUS rats [one-way ANOVA, *F*(4,34) = 3.993, *p* = 0.009; Dunnett’s test, *p* < 0.05], demonstrating the predictive validity of CUS model. Chronic oral administration of CTE in high dose group showed key effects on sucrose preference [one-way ANOVA, *F*(4,34) = 3.993, *p* = 0.009; Dunnett’s test, *p* < 0.01], which restored it to normal levels in CUS rats. CTE with low dose group showed an increasing trend but the effect was not statistically significant (Dunnett’s test, *p* = 0.073).

As shown in **Figure 1C**, total distance covered in OFT was also used to evaluate the effects of CTE on CUS rats. Compared with non-stress rats, CUS model group manifested a much shorter total distance [one-way ANOVA, *F*(4,34) = 7.845, *p* = 0.0001; Dunnett’s test, *p* < 0.001] after a 4-week exposure to CUS, and the positive control FLX treatment increased the total distance in CUS rats [one-way ANOVA, *F*(4,34) = 7.845, *p* = 0.0001; Dunnett’s test, *p* < 0.05]. Daily oral administration of CTE with high dose and low dose showed an evident effect on the total distance in CUS rats [one-way ANOVA, *F*(4,34) = 7.845, *p* = 0.0001; Dunnett’s test, *p* < 0.01 for high dose group, *p* < 0.001 for low dose group, respectively]. **Supplementary Figure S4** shows the activity track map in OFT.

**Figure 1D** shows the effects of CTE on latency to eat in the NSFT in CUS rats. Twenty-eight days stress procedure caused a significant increase in latency to begin eating in the CUS model group compared to the control non-stressed rats [one-way ANOVA, *F*(4,33) = 3.434, *p* = 0.019; Dunnett’s test, *p* < 0.05], indicating that the CUS model was successfully developed. Long-term oral administration of CTE with high dose showed evident effects on latency to begin eating compared with the CUS group (Student’s *t*-test, *t* = 2.759, *p* < 0.05), while low dose of CTE showed no effect.

### CTE Restored the Level of Neurotransmitters and Neurotrophic Factors in CUS Rats

**FIGURE 2:**
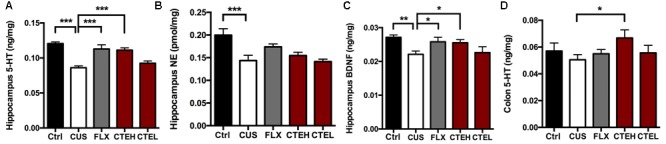
The changes of monoamine neurotransmitter levels in hippocampus and colon of CUS rats treated with CTE: 5-HT **(A)**, NE **(B)**, and BDNF **(C)** in hippocampus, and 5-HT in colon **(D)**. Ctrl, control; CUS, chronic unpredictable stress; FLX, fluoxetine; CTEH, *C. tubulosa* extract high dose; CTEL, *C. tubulosa* extract low dose. ^∗^*P* < 0.05, ^∗∗^*P* < 0.01, ^∗∗∗^*P* < 0.001 (*n* = 8, mean ± SEM).

The effects of CTE on 5-HT and NE levels in the hippocampus of CUS rats is shown in **Figures 2A,B**. Four weeks stress procedure caused a significant reduction of 5-HT [one-way ANOVA, *F*(4,34) = 17.13, *p* = 0.0001; Dunnett’s test, *p* < 0.001] and NE [one-way ANOVA, *F*(4,34) = 6.376, *p* = 0.0006; Dunnett’s test, *p* < 0.001] concentration in the hippocampus of CUS rats compared with that in control group, and the antidepressant FLX significantly restored 5-HT levels in the hippocampus [one-way ANOVA, *F*(4,34) = 17.13, *p* = 0.0001; Dunnett’s test, *p* < 0.001]. After oral administration of CTE in high dose group, significant increase in 5-HT level was observed [one-way ANOVA, *F*(4,34) = 17.13, *p* = 0.0001; Dunnett’s test, *p* < 0.001] while the NE concentrations were not changed. Similarly, BDNF expression was significantly reduced in the CUS model group compared with the non-stressed rats (Student’s *t*-test, *t* = 4.171, *p* < 0.01) (**Figure 2C**). Both long-term oral administration of CTE with high dose (Student’s *t*-test, *t* = 2.548, *p* < 0.05) and FLX (Student’s *t*-test, *t* = 2.263, *p* < 0.05) increased BDNF expression compared with CUS model group. CTE with low dose showed no significant effect on BDNF expression.

The 5-HT level in colon of CUS rats was different from that in the hippocampus, and 4-week stress procedure had no impact on colon 5-HT (**Figure 2D**). Conversely, oral administration of CTE with high dose stimulated 5-HT levels in the colon when compared with CUS model group (Student’s *t*-test, *t* = 2.173, *p* < 0.05). CTE with low dose showed no effect on colon 5-HT level.

### CTE Regulated the Gut Microbial Composition of CUS Rats

The effect of CTE on gut microbial composition of CUS rat was evaluated by the 16S rRNA gene sequencing-based method. It was found that 28 days oral administration of CTE altered gut microbial composition of CUS rats and impacted various microbial taxonomic levels among rat groups (class level 1, order level 1, family level 1, genus level 8, and species level 2). This suggests that the gut microbial community could play a role in the anti-depressant effects of CTE. Detailed information of significantly altered gut microbial taxa at different taxonomic levels is shown in **Table 2**.

**Table 2:** Significantly altered gut microbial taxa at different taxonomic levels in chronic unpredictable stress rats treated with *C. tubulosa* extract.

No.	Phylum	Class	Order	Family	Genus	Species
1	Bacteroidetes	Bacteroidia	Bacteroidales	Bacteroidaceae	*Bacteroides*^∗^ (↘↗)^a^	
2	Bacteroidetes	Bacteroidia	Bacteroidales	Porphyromonadaceae	*Parabacteroides*^∗^ (↘↗)^a^	
3	Bacteroidetes	Bacteroidia	Bacteroidales	[Odoribacteraceae]	*Butyricimonas*^∗^ (↘↗)^a^	
4	[Thermi]	Deinococci^∗^ (↗↘)^a^	Deinococcales^∗^ (↗↘)^a^	Deinococcaceae^∗^ (↗↘)^a^	*Deinococcus*^∗^ (↗↘)^a^	
5	Firmicutes	Bacilli	Lactobacillales	Leuconostocaceae	*Weissella*^∗^ (↘↗)^a^	*Weissella beninensis*^∗^ (↘↗)^a^
6	Firmicutes	Bacilli	Lactobacillales	Carnobacteriaceae	*Trichococcus*^∗^ (↗↘)^a^	
7	Firmicutes	Clostridia	Clostridiales	Ruminococcaceae	*Ruminococcus*^∗^ (↗↘)^a^	
8	Actinobacteria	Actinobacteria	Actinomycetales	Dermabacteraceae	*Brachybacterium*^∗^ (↗↘)^a^	*Brachybacterium conglomeratum*^∗^ (↗↘)^a^

*^∗^Significantly altered gut microbial taxa. ^*a*^First slanted arrow means CUS model group compared with control group, second slanted arrow means CTE treated group (high or low dose) compared with CUS model group.*

**FIGURE 3:**
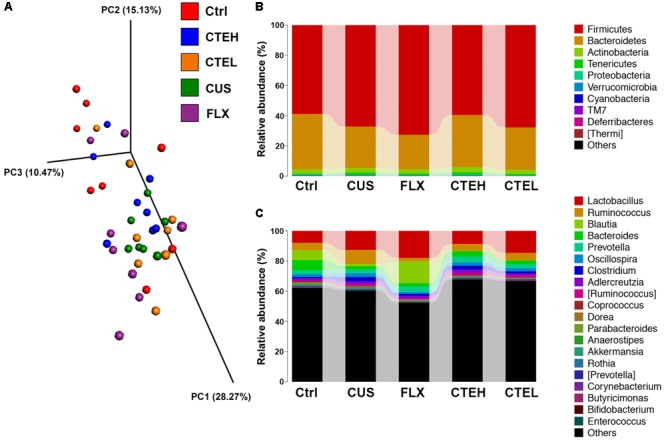
3D diagram of Weighted UniFrac PCoA of intestinal microbiota extracted from rat fecal samples **(A)**. Microbial distribution at phylum level **(B)**. Microbial distribution at genus level **(C)**. Ctrl, control; CUS, chronic unpredictable stress; FLX, fluoxetine; CTEH, *C. tubulosa* extract high dose; CTEL, *C. tubulosa* extract low dose.

The MiSeq sequencing yielded a total of 2,132,980 raw reads, following quality control, denoising, and chimera removal process. Next, the reads were clustered into OTUs, which were assigned to taxa from phylum to species level. The study of beta diversity analysis was performed to explore the similarity of bacterial community patterns among the five rat groups. PCoA based on weighted UniFrac distances of gut microbiota from the five rat groups of study is shown in **Figure 3A**. Beta diversity analysis by PCoA showed a clear separation of the microbial community from the control group and the CUS model group, while the CTE with high dose group trended to be closer to the control group as compared with the CUS group.

Metastats analysis was used to investigate the differences in gut microbial taxonomic composition among different groups. At phylum level, the most dominant microbial taxa in the five rat groups were Firmicutes and Bacteroidetes (**Figure 3B**). *Lactobacillus, Ruminococcus, Blautia, Bacteroides*, and *Prevotella* were the most highly abundant microbial taxa at the genus level (**Figure 3C**). The two dominant phyla, Firmicutes and Bacteroidetes, accounted for a combined relative abundance of >90%. As shown in **Supplementary Figures S5A,B**, 4 weeks stress procedure caused a non-significant trend toward an increased level of Firmicutes and a non-significant decrease of Bacteroidetes in the CUS model group as compared to the control group. After daily administration of CTE with high dose and low dose, the relative abundance of Firmicutes and Bacteroidetes returned to the similar levels of the control group, though with no statistical significance. And although similar changes were observed for Firmicutes/Bacteroidetes ratio among the rat groups, no statistical differences were shown (**Supplementary Figure S5C**). A non-significant trend toward a decreased level of phyla Cyanobacteria was found in the CUS model group compared to the control non-stressed rats; whereas administration of CTE with high dose resulted in a significant increase in the relative abundance of Cyanobacteria compared with the CUS group (**Supplementary Figure S5D**).

**FIGURE 4:**
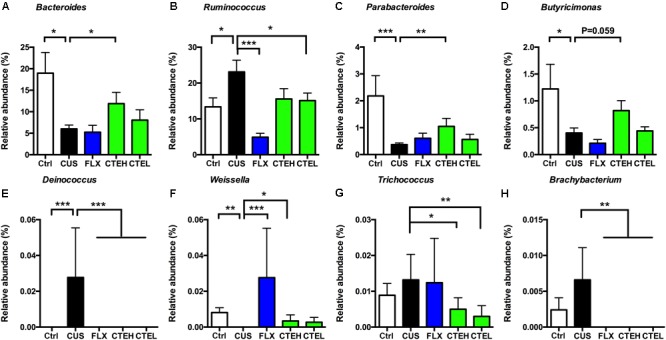
Relative abundance of selected genera with significant differences among each group. Relative abundance of *Bacteroides* **(A)**, *Ruminococcus* **(B)**, *Parabacteroides* **(C)**, *Butyricimonas* **(D)**, *Deinococcus* **(E)**, *Weissella* **(F)**, *Trichococcus* **(G)**, and *Brachybacterium* **(H)**. Ctrl, control; CUS, chronic unpredictable stress; FLX, fluoxetine; CTEH, *C. tubulosa* extract high dose; CTEL, *C. tubulosa* extract low dose. ^∗^*P* < 0.05, ^∗∗^*P* < 0.01, ^∗∗∗^*P* < 0.001 (*n* = 8, mean ± SEM).

At genus level, *Bacteroides* and *Ruminococcus* were two of the most highly abundant microbial taxa that accounted for approximately 30% relative abundance in all five rat groups. Four weeks stress procedure caused a significant reduction in relative abundance of *Bacteroides* and a significant increase of *Ruminococcus* in the CUS model group as compared to the control non-stressed rats (**Figures 4A,B**). Long-term administration of CTE resulted in a significant increase in the abundance of *Bacteroides* as well as a significant decrease of *Ruminococcus* compared to the CUS rats. Additionally, six genera of *Parabacteroides, Butyricimonas, Deinococcus, Weissella, Trichococcus*, and *Brachybacterium* showed statistically significant differences between the CUS model group and the control group (**Figures 4C–H**). After 28 days treatment of CTE, the relative abundance of abovementioned six genera was respectively changed to levels similar to the control group.

**FIGURE 5:**
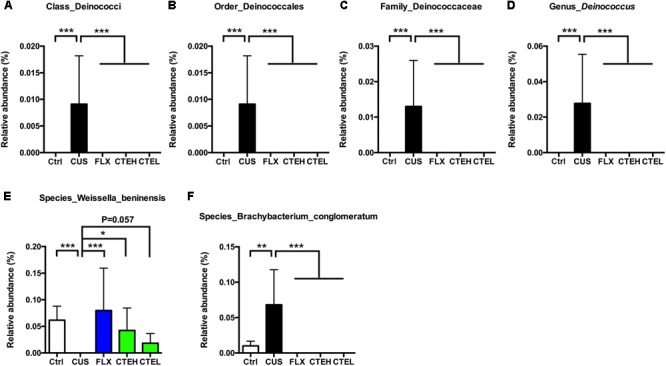
Relative abundance of *Deinococcus* in different taxonomic levels: **(A)** class level, **(B)** order level, **(C)** family level, **(D)** genus level. Relative abundance of selected species with significant differences among each group. Relative abundance of *Weissella beninensis* **(E)**, and *Brachybacterium conglomeratum* **(F)**. Ctrl, control; CUS, chronic unpredictable stress; FLX, fluoxetine; CTEH, *C. tubulosa* extract high dose; CTEL, *C. tubulosa* extract low dose. ^∗^*P* < 0.05, ^∗∗^*P* < 0.01, ^∗∗∗^*P* < 0.001 (*n* = 8, mean ± SEM).

Low abundance of *Deinococcus* in different taxonomic levels including class level (Deinococci), order level (Deinococcales), family level (Deinococcaceae), and genus level (*Deinococcus*) was detected in the CUS rat group, while it failed to be detected in the control group and the administration groups (**Figures 5A–D**).

At the species level, *Weissella beninensis* was much lower in the CUS group than in the control group, and a significant increase in relative abundance was observed after administration of CTE with high dose (**Figure 5E**). Conversely, CTE administration reversed the significant increase in relative abundance of *Brachybacterium conglomeratum* observed in the CUS rats (**Figure 5F**).

### CTE Modulated SCFAs in CUS Rats

**FIGURE 6:**
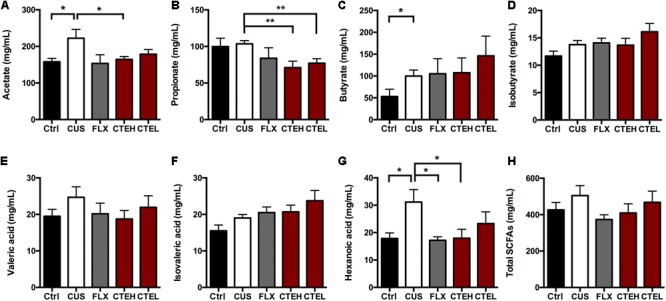
The changes of SCFAs in fecal samples of CUS rats treated with CTE: **(A)** acetate, **(B)** propionate, **(C)** butyrate, **(D)** isobutyrate, **(E)** valeric acid, **(F)** isovaleric acid, **(G)** hexanoic acid, and **(H)** total SCFAs. Ctrl, control; CUS, chronic unpredictable stress; FLX, fluoxetine; CTEH, *C. tubulosa* extract high dose; CTEL, *C. tubulosa* extract low dose. ^∗^*P* < 0.05, ^∗∗^*P* < 0.01 (*n* = 8, mean ± SEM).

The SCFAs concentrations (including acetate, propionate, butyrate, isobutyrate, valeric acid, isovaleric acid, and hexanoic acid) in rat fecal samples were analyzed by GC and are shown in **Figure 6**. The levels of acetate [one-way ANOVA, *F*(4,32) = 2.721, *p* = 0.0467; Dunnett’s test, *p* < 0.05] and hexanoic acid [one-way ANOVA, *F*(4,30) = 3.028, *p* = 0.0328; Dunnett’s test, *p* < 0.05] were significantly increased in the CUS model group rats compared to the control group. After long-term administration of CTE with high dose, acetate (Student’s *t*-test, *t* = 2.182, *p* < 0.05) and hexanoic acid [one-way ANOVA, *F*(4,30) = 3.028, *p* = 0.0328; Dunnett’s test, *p* < 0.05] concentrations were predominantly decreased when compared with CUS group, indicating CTE might exert its anti-depressant activity via modulating the disordered neuroactive metabolite SCFAs. Administration of CTE with low dose group showed a decreasing trend but no remarkable effect on acetate and hexanoic acid concentrations.

### Correlation Analysis of the Gut Microbiota, Neurotransmitters and Neurotrophins in Hippocampus, 5-HT in Colon, and SCFAs

**FIGURE 7:**
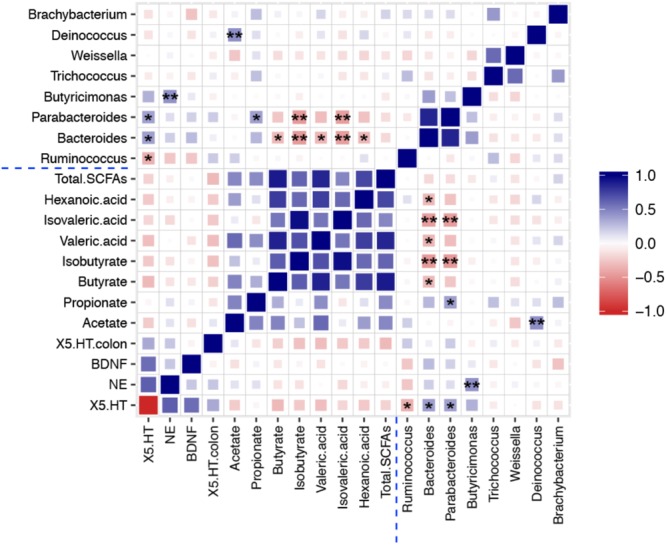
Heat map summarizing the Pearson’s correlation between 5-HT, NE, and BDNF in hippocampus, 5-HT in colon, gut microbiota, and SCFAs. ^∗^*P* < 0.05, ^∗∗^*P* < 0.01.

To explore the functional relationship of altered gut microbiota in genus level, changed neurotransmitters and neurotrophins in hippocampus and colon, and the disturbed concentration of SCFAs, Pearson’s correlation coefficients were developed (Meng et al., 2017). Correlations between them (*r* > 0.4 or *r* < -0.4, *p* < 0.05) are shown in **Figure 7**. The coefficients indicated strong correlations between altered gut microbiota composition in genus level, the concentration of seven types of SCFAs, and depression-related monoamine neurotransmitters (5-HT and NE). The abundance of *Ruminococcus* showed significant negative correlation with 5-HT concentration in the hippocampus, which means that CUS procedure leads to an increment in the relative abundance of *Ruminococcus* and decrement in 5-HT concentrations. After treatment with CTE, the relative abundance of *Ruminococcus* was reduced to the level of control group, and the 5-HT levels were found to have improved. Further, the relative abundance of *Bacteroides* was positively correlated with 5-HT level in the hippocampus, and negatively associated with the concentration of butyrate, isobutyrate, valeric acid, isovaleric acid, and hexanoic acid; the abundance of *Parabacteroides* displayed a significant positive correlation with 5-HT level in the hippocampus and propionate concentration, while it showed a negative correlation with isobutyrate and isovaleric acid concentrations; a significantly positive association between the relative abundance of *Butyricimonas* and NE level in the hippocampus was also observed; and the relative abundance of *Deinococcus* was positively correlated with acetate concentration. This demonstrated that CTE might exert its anti-depressant activity by changing the composition of the gut microbiota, disturbing hippocampus neurotransmitter levels, and restoring neuroactive metabolites SCFAs. Detailed data of correlation analysis is shown in **Supplementary Table S3**.

## Discussion

### Identification of Anti-depression Activity of CTE and Its Clinical Advantages

This study evaluated the antidepressant activity of CTE in CUS rats, and confirmed the efficacy in *in vivo* models such as FST, SPT, OFT, and NSFT, and confirmed the anti-depressive activity of *C. tubulosa* in CUS rats which is frequently used for the treatment of kidney deficiency, impotence, and female infertility in TCM. Currently, the most commonly prescribed antidepressants are the serotonin and norepinephrine reuptake inhibitor (SNRI) venlafaxine, followed by the selective serotonin reuptake inhibitor (SSRI) FLX (Thase et al., 2001; Mcintyre, 2017). However, these antidepressants have been shown to possess severe side effects including cardiac toxicity, blood pressure, sexual dysfunction, and sleep disorders (Ferguson, 2001; Jin et al., 2015). Among all classes of antidepressants, SSRI and SNRI are correlated with the highest incidence of sexual dysfunction (Montejogonzález et al., 1997; Clayton et al., 2014). Of particular note is the overall incidence of sexual dysfunction, which is at 59.1% (604/1,022) when all antidepressants are considered as a whole; and about 40% of patients show low tolerance to sexual dysfunction, thus drug compliance is severely affected (Clayton, 2001). In view of the drawbacks of current depression treatment, Cistanches Herba shows great potential for clinical application, not only because of its potent antidepressant-like activity, but also because Cistanches Herba has been traditionally used to treat impotence, which should at least eliminate one concern of the most common side effects caused by other antidepressants (Fu et al., 2017). In addition, Cistanches Herba shows no signs of toxicity and treatment-related changes in rats, makes us believe it has less harmful side effects and is very safe when used therapeutically (Gao et al., 2016).

### CTE Modulates Neurotransmitters and Neurotrophins

Results from this study indicate that CTE administration can significantly increase CUS-induced hippocampus 5-HT level and BDNF expression, along with 5-HT level in the colon. This could well explain the antidepressant activates that CTE exerts. More than 90% of 5-HT in the body is synthesized by specialized endocrine cells in the gut called enterochromaffin cells (Gershon and Tack, 2007; Yano et al., 2015). Previous research demonstrated that spore-forming bacteria in mouse and human microbiota promoted 5-HT biosynthesis from colonic enterochromaffin cells, which supply 5-HT to the mucosa, lumen, and circulating platelets for hosting 5-HT regulation (Yano et al., 2015). So it would be critical to clarify the association between altered gut microbiota structure and 5-HT concentration in colon after oral administration of CTE, which will shed light upon the pharmacological mechanism of action for the antidepressant-like activity of CTE. Although CTE administration led to significant gut microbiota changes and 5-HT amount increases in the colon, no correlation was found between the altered gut microbiota and the changed 5-HT level in the colon based on Pearson’s correlation coefficients. Therefore, it remains unclear whether the homeostasis of gut microbiota led to 5-HT biosynthesis in the colon after CTE administration of the CUS rats. Our ongoing experiment of microbiota transplantation in germ-free mice is to further investigate this.

It has been reported that the aqueous extract of *C. tubulosa* exhibited an antidepressant effect in the mouse model, modulation of the monoamine system and HPA axis both contributes to the antidepressant effect of *C. tubulosa* (Wang D. et al., 2017). In our present study, CTE significantly improved depression-like behaviors in rats under CUS by regulating neurotransmitters and neurotrophins in hippocampus. This well-defined extract, which is composed by 48.6% PhGs, 6.9% iridoid glycosides, and 20.0% total saccharides suggesting this mixture might exert the antidepressant effect through multiple ways. For example, PhGs can increasing DA levels in the striatum (Tian and Pu, 2005; Geng et al., 2007), iridoids can restoring HPA axis dysfunctions and upregulating BDNF expression (Cai et al., 2015; Wang et al., 2015). However, the exact target of the antidepressant effect of CTE on the molecular level, and the contributions from the main constituents in the extract remains unknown, further study is needed to elucidate the comprehensive antidepressant mechanism of CTE.

### CTE Restores Gut Microbiota Composition and SCFAs Production

In this study, the homeostasis of gut microbiota composition was restored by CTE in the CUS model. An abundance of *Bacteroides*, strict anaerobes with high importance in the gut from early-life (Arboleya et al., 2015), was elevated after CTE administration. Previous studies indicate that *Bacteroides fragilis* could reverse autism-like behaviors in mice (Hsiao et al., 2013). Our results demonstrate that *Bacteroides* was positively associated with 5-HT in the hippocampus, and negatively associated with SCFAs, which is correlated with the observed anti-depressive effect of CTE. Moreover, changes in microbial composition have recently been linked to host immunity (Round and Mazmanian, 2009). For example, various *Bacteroides* spp. can expand T_reg_ cell population, bias the T_H_1/T_H_2 phenotype, or suppress host inflammatory responses by SCFAs (Samuelson et al., 2015). The immune system provides an additional connection between gut microbiota and depression (Miller et al., 2009). Hence, whether CTE affects *Bacteroides* population, further regulates the immune system, and then exert its anti-depressive effects on the host, needs to be investigated in the future. A previous publication indicated prebiotic administration resulted in significant increase in the abundance of *Bacteroides* and *Parabacteroides*, and a decrease in the abundance of *Ruminococcus* (Burokas et al., 2017). CTE administration also led to similar increase of *Bacteroides* and *Parabacteroides*, as well as decrease of *Ruminococcus*. This is probably because the oligosaccharides and polysaccharides in CTE are potential sources of prebiotics. Low abundance of *Deinococcus* was only detected in the CUS-induced model group, but not detected in control and CTE administration group. This suggests that the role of these non-dominant bacteria in depression should not be ignored.

*Deinococcus* was first discovered in 1956, and is known for its remarkable resistance to damage caused by a range of factors—ionizing radiation, desiccation, UV radiation, and oxidizing agents (Gerber et al., 2015). Additionally, *Deinococcus* possesses the ability to degrade and metabolize sugars due to the presence of genes encoding sugar-metabolizing enzymes (Gerber et al., 2015). In this study, a dramatic increase in relative abundance of *Deinococcus* was observed in CUS-induced group, and following CTE administration the level of *Deinococcus* dropped significantly to the level of the control group. This implies that CUS might lead to a disordered sugar metabolism level in gut microbiota, and increase in *Deinococcus* might attribute to the stress response in CUS rats. After long-term treatment of CTE, CUS rat’s sugar metabolism levels could become normal, and the abnormal growth of *Deinococcus* would then disappear. Based on this, non-dominant bacteria such as the genus *Deinococcus* can serve as a diagnostic marker for the onset of depression and demonstrate the practicability for the study of the regulation of gut microbiota as a therapeutic target.

Many species from genus *Weissella* have been isolated and used as probiotic lactic acid bacteria (LAB) due to their beneficial anti-inflammatory, immunomodulatory, and anti-oxidation effects (Ojekunle et al., 2017; Park et al., 2017; Sandes et al., 2017). There is no publication so far that focuses on the connection between the genus *Weissella* and depression. Our results show that, 28 days stress procedure caused a significant reduction in the relative abundance of *Weissella* in the CUS model group compared to the control non-stressed rats, and daily oral administration of CTE caused a significant increase on the relative abundance of *Weissella* compare to CUS rats. Interestingly, *W. beninensis* at species level showed consistent results as *Weissella* did at genus level. This confirms a substantial relationship between *Weissella* and the application of CTE for treatment of depression. Thus, the combinational use of *Weissella* probiotics and CTE would be even more beneficial for depression patients.

Short-chain fatty acids are the key molecules that modulate microglia maturation and function, as well as depression (Erny et al., 2015; Dinan and Cryan, 2017). In this study, it was found that CTE could reverse the disordered concentration of acetate and hexanoic acid to a reasonable level. Previous research demonstrated that acetate directly interacts with the hypothalamic mechanisms in the brain (Frost et al., 2014), implying that CTE could regulate disordered acetate and HPA axis for anti-depression. However, there is no published study focusing on the association between hexanoic acid and depression development, which we will further investigate in future.

## Conclusion

In conclusion, CTE exerted potent antidepressant activities via restoring the level of 5-HT, BDNF, and SCFAs, and modulating the relative abundance of gut microbiota in genus level in CUS rats. Correlation analysis revealed that altered gut microbiota genera were also substantially with changed neurotransmitters, neurotrophins, and SCFAs levels. Therefore, CTE was identified as a potential therapeutic agent for depression targeting the microbiota–gut–brain axis.

## Data Availability

All raw sequences were deposited in the NCBI Sequence Read Archive under accession number SRP128788. The authors declare that all other data supporting the findings of this study are available within the article and its **Supplementary Material**, or are available from the corresponding author on request with no restrictions.

## Author Contributions

YL, YP, PT, and XL designed the experiments. YL, PM, HY, and HX carried out the experiments. YL, YP, MW, CP, and XL analyzed the data. YL, YP, and XL wrote the manuscript.

## Conflict of Interest Statement

The authors declare that the research was conducted in the absence of any commercial or financial relationships that could be construed as a potential conflict of interest.
