# Supplementary material for: Antidepressant-Like Effects of Cistanche tubulosa Extract on Chronic Unpredictable Stress Rats Through Restoration of Gut Microbiota Homeostasis
Source: Front Pharmacol. 2018 Aug 21;9:967. doi: 10.3389/fphar.2018.00967 (PMC6112285; doi:10.3389/fphar.2018.00967)
Supplement: Supplementary file 1 [file Table_1.DOCX]

**Supplementary Material**

**Table S1** Chronic unpredictable stress regime.

| Day | Week |  |  |  |
| --- | --- | --- | --- | --- |
|  | Week 1 | Week 2 | Week 3 | Week 4 |
| Sunday | White noise: 1 h  Overnight stroboscope: 12 h | Cage tilt: 24 h | Cage tilt: 24 h | Cage tilt: 24 h |
| Monday | Forced swimming: 15 min  (Forced swimming pretest) | Shock: 30 min | Shock: 30 min | Forced swimming: 5 min |
| Tuesday | Restraint: 1 h  Overnight illumination: 12 h | White noise: 2 h  Overnight illumination: 12 h | Water depravation: 24 h | Shock: 30 min |
| Wednesday | Forced swimming: 5 min  (Forced swimming test) | Restraint: 2 h  Overnight stroboscope: 12 h | Tail pinch: 1 min | Soiled cage: 24 h |
| Thursday | Soiled cage: 24 h | Soiled cage: 24 h | Soiled cage: 24 h | Tail pinch: 1 min |
| Friday | Tail pinch: 1 min | Forced swimming: 5 min | Restraint: 2 h  Overnight stroboscope: 12 h | Food depravation: 24 h |
| Saturday | Water depravation: 24 h | Food depravation: 24 h | Food depravation: 24 h | Cage tilt: 24 h |

**Table S2** Characterization of chemical constituents of *C. tubulosa* extract by UPLC-Q-TOF-MS.

| No. | t_R_ (min) | Measured mass (Da) | Error (mDa) | Formula | MS/MS fragment ions (Da) | Identification |
| --- | --- | --- | --- | --- | --- | --- |
| C1 | 1.90 | 649.1978 | −0.2 | C_27_H_38_O_18_ | 179.0349, 135.0451 | kankanose |
| C2 | 2.00 | 375.1292 | 0.1 | C_16_H_24_O_10_ | 151.0764 | 8-epiloganic acid or isomer |
| C3 | 2.09 | 461.1661 | 0.2 | C_20_H_30_O_12_ | 135.0450 | decaffeoylacteoside |
| C4 | 2.23 | 487.1449 | −0.3 | C_21_H_28_O_13_ | 179.0346, 135.0449 | cistanoside F |
| C5 | 2.85 | 375.1294 | 0.3 | C_16_H_24_O_10_ | 167.0713 | 8-epiloganic acid or isomer |
| C6 | 3.36 | 801.2448 | −0.3 | C_35_H_46_O_21_ | 623.2181, 179.0350, 161.0244, 135.0451 | cistantubuloside C_1_/C_2_ |
| C7 | 3.58 | 503.1766 | −0.1 | C_22_H_32_O_13_ | 431.1187 | cistanoside H |
| C8 | 4.09 | 639.1924 | −0.1 | C_29_H_36_O_16_ | 179.0348, 161.0244, 135.0451 | campneoside II |
| C9 | 4.22 | 785.2500 | −0.4 | C_35_H_46_O_20_ | 623.2185, 477.1610, 315.1082, 161.0243 | echinacoside |
| C10 | 4.54 | 639.1920 | −0.5 | C_29_H_36_O_16_ | 179.0350, 161.0244, 135.0449 | isomer of campneoside II |
| C11 | 4.69 | 769.2551 | −0.4 | C_35_H_46_O_19_ | 161.0243 | poliumoside |
| C12 | 4.74 | 769.2550 | −0.5 | C_35_H_46_O_19_ | 623.2185, 135.0451 | isopoliumoside |
| C13 | 4.89 | 799.2647 | −1.4 | C_36_H_48_O_20_ | 623.2178 | cistanoside A |
| C14 | 5.05 | 345.1549 | 0.0 | C_16_H_26_O_8_ | 135.0451 | kankanoside A or isomer |
| C15 | 5.31 | 827.2600 | −1.0 | C_37_H_48_O_21_ | 785.2490, 665.2288, 623.2006, 461.1662, 315.1079, 161.0242 | tubuloside A |
| C16 | 5.32 | 623.1972 | −0.4 | C_29_H_36_O_15_ | 461.1662, 315.1079, 161.0242 | acteoside |
| C17 | 5.45 | 345.1550 | 0.1 | C_16_H_26_O_8_ | 161.0242, 135.0450 | kankanoside A or isomer |
| C18 | 5.71 | 623.1969 | −0.7 | C_29_H_36_O_15_ | 461.1659, 315.1081, 179.0347, 161.0241 | isoacteoside |
| C19 | 5.92 | 347.1706 | 0.0 | C_16_H_28_O_8_ | − | kankanoside N |
| C20 | 6.26 | 637.2121 | −1.1 | C_30_H_38_O_15_ | − | cistanoside C |
| C21 | 6.46 | 665.2077 | −0.5 | C_31_H_38_O_16_ | 461.1656, 161.0241 | 2′-acetylacteoside |
| C22 | 6.62 | 591.2078 | 0.0 | C_29_H_36_O_13_ | − | osmanthuside B or osmanthuside B6 |
| C23 | 6.75 | 637.2123 | −0.9 | C_30_H_38_O_15_ | − | isocistanoside C |
| C24 | 6.93 | 665.2081 | −0.1 | C_31_H_38_O_16_ | 623.1993, 503.1763, 461.1665, 315.1080, 179.0350, 161.0243 | tubuloside B |
| C25 | 7.06 | 591.2082 | 0.4 | C_29_H_36_O_13_ | − | osmanthuside B or osmanthuside B6 |
| C26 | 7.08 | 649.2134 | 0.2 | C_31_H_38_O_15_ | − | salsaside F or isomer |
| C27 | 7.59 | 649.2130 | −0.2 | C_31_H_38_O_15_ | − | salsaside F or isomer |

**Table S3** Pearson’s correlation matrix between 5-HT, NE, BDNF in brain, 5-HT in colon, gut microbiota, and SCFAs.

| Name | 5-HT | NE | BDNF | Colon 5-HT | acetate | propionate | butyrate | isobutyrate | valeric acid | isovaleric acid | hexanoic acid | total SCFAs | G1 | G2 | G3 | G4 | G5 | G6 | G7 | G8 |
| --- | --- | --- | --- | --- | --- | --- | --- | --- | --- | --- | --- | --- | --- | --- | --- | --- | --- | --- | --- | --- |
| 5-HT | 1 | 0.63 | 0.57 | 0.33 | -0.24 | -0.04 | -0.32 | -0.25 | -0.30 | -0.19 | -0.25 | -0.21 | -0.36^*^ | 0.37^*^ | 0.37^*^ | 0.30 | -0.09 | 0.00 | -0.13 | -0.14 |
| NE | 0.63 | 1 | 0.22 | 0.22 | 0.11 | 0.12 | -0.13 | -0.14 | -0.01 | -0.18 | -0.07 | -0.07 | -0.26 | 0.19 | 0.13 | 0.43^**^ | -0.06 | 0.05 | 0.03 | -0.04 |
| BDNF | 0.57 | 0.22 | 1 | -0.06 | -0.14 | 0.04 | -0.11 | -0.05 | -0.13 | 0.01 | -0.06 | 0.02 | -0.26 | 0.27 | 0.18 | 0.08 | -0.11 | 0.01 | -0.13 | -0.28 |
| Colon 5-HT | 0.33 | 0.22 | -0.06 | 1 | 0.09 | -0.12 | -0.22 | -0.29 | -0.30 | -0.25 | -0.24 | -0.32 | 0.21 | 0.06 | 0.20 | -0.06 | 0.07 | -0.09 | 0.05 | -0.10 |
| acetate | -0.24 | 0.11 | -0.14 | 0.09 | 1 | 0.48 | 0.49 | 0.24 | 0.59 | 0.08 | 0.38 | 0.47 | 0.19 | 0.01 | -0.05 | -0.02 | -0.08 | -0.26 | 0.44^**^ | 0.05 |
| propionate | -0.04 | 0.12 | 0.04 | -0.12 | 0.48 | 1 | 0.32 | 0.08 | 0.44 | -0.11 | 0.12 | 0.46 | -0.04 | 0.28 | 0.37^*^ | -0.02 | 0.25 | 0.11 | 0.12 | 0.26 |
| butyrate | -0.32 | -0.13 | -0.11 | -0.22 | 0.49 | 0.32 | 1 | 0.64 | 0.87 | 0.53 | 0.77 | 0.90 | 0.03 | -0.32^*^ | -0.26 | -0.03 | 0.02 | -0.16 | 0.01 | 0.06 |
| isobutyrate | -0.25 | -0.14 | -0.05 | -0.29 | 0.24 | 0.08 | 0.64 | 1 | 0.68 | 0.95 | 0.59 | 0.61 | -0.05 | -0.46^**^ | -0.47^**^ | -0.07 | -0.01 | -0.12 | -0.08 | 0.03 |
| valeric acid | -0.30 | -0.01 | -0.13 | -0.30 | 0.59 | 0.44 | 0.87 | 0.68 | 1 | 0.54 | 0.75 | 0.85 | 0.00 | -0.33^*^ | -0.30 | -0.02 | 0.03 | -0.13 | 0.10 | 0.16 |
| isovaleric acid | -0.19 | -0.18 | 0.01 | -0.25 | 0.08 | -0.11 | 0.53 | 0.95 | 0.54 | 1 | 0.59 | 0.47 | -0.02 | -0.45^**^ | -0.45^**^ | 0.01 | -0.12 | -0.16 | -0.14 | -0.08 |
| hexanoic acid | -0.25 | -0.07 | -0.06 | -0.24 | 0.38 | 0.12 | 0.77 | 0.59 | 0.75 | 0.59 | 1 | 0.72 | 0.12 | -0.32^*^ | -0.28 | 0.01 | -0.11 | -0.19 | 0.16 | -0.07 |
| total SCFAs | -0.21 | -0.07 | 0.02 | -0.32 | 0.47 | 0.46 | 0.90 | 0.61 | 0.85 | 0.47 | 0.72 | 1 | -0.01 | -0.18 | -0.16 | -0.10 | 0.07 | -0.13 | -0.03 | 0.17 |
| G1 | -0.36^*^ | -0.26 | -0.26 | 0.21 | 0.19 | -0.04 | 0.03 | -0.05 | 0.00 | -0.02 | 0.12 | -0.01 | 1 | 0.01 | -0.11 | 0.02 | 0.26 | -0.19 | 0.09 | 0.14 |
| G2 | 0.37^*^ | 0.19 | 0.27 | 0.06 | 0.01 | 0.28 | -0.32^*^ | -0.46^**^ | -0.33^*^ | -0.45^**^ | -0.32^*^ | -0.18 | 0.01 | 1 | 0.86 | 0.39 | -0.13 | -0.10 | -0.10 | 0.13 |
| G3 | 0.37^*^ | 0.13 | 0.18 | 0.20 | -0.05 | 0.37^*^ | -0.26 | -0.47^**^ | -0.30 | -0.45^**^ | -0.28 | -0.16 | -0.11 | 0.86 | 1 | 0.25 | -0.08 | -0.06 | -0.08 | 0.13 |
| G4 | 0.30 | 0.43^**^ | 0.08 | -0.06 | -0.02 | -0.02 | -0.03 | -0.07 | -0.02 | 0.01 | 0.01 | -0.10 | 0.02 | 0.39 | 0.25 | 1 | -0.15 | -0.19 | -0.01 | 0.01 |
| G5 | -0.09 | -0.06 | -0.11 | 0.07 | -0.08 | 0.25 | 0.02 | -0.01 | 0.03 | -0.12 | -0.11 | 0.07 | 0.26 | -0.13 | -0.08 | -0.15 | 1 | 0.59 | 0.00 | 0.40 |
| G6 | 0.00 | 0.05 | 0.01 | -0.09 | -0.26 | 0.11 | -0.16 | -0.12 | -0.13 | -0.16 | -0.19 | -0.13 | -0.19 | -0.10 | -0.06 | -0.19 | 0.59 | 1 | -0.05 | -0.04 |
| G7 | -0.13 | 0.03 | -0.13 | 0.05 | 0.44^**^ | 0.12 | 0.01 | -0.08 | 0.10 | -0.14 | 0.16 | -0.03 | 0.09 | -0.10 | -0.08 | -0.01 | 0.00 | -0.05 | 1 | -0.05 |
| G8 | -0.14 | -0.04 | -0.28 | -0.10 | 0.05 | 0.26 | 0.06 | 0.03 | 0.16 | -0.08 | -0.07 | 0.17 | 0.14 | 0.13 | 0.13 | 0.01 | 0.40 | -0.04 | -0.05 | 1 |

G1: *Ruminococcus*, G2: *Bacteroides*, G3: *Parabacteroides*, G4: *Butyricimonas*, G5: *Trichococcus*, G6: *Weissella*, G7: *Deinococcus*, G8: *Brachybacterium*; *P<0.05, **P<0.01.


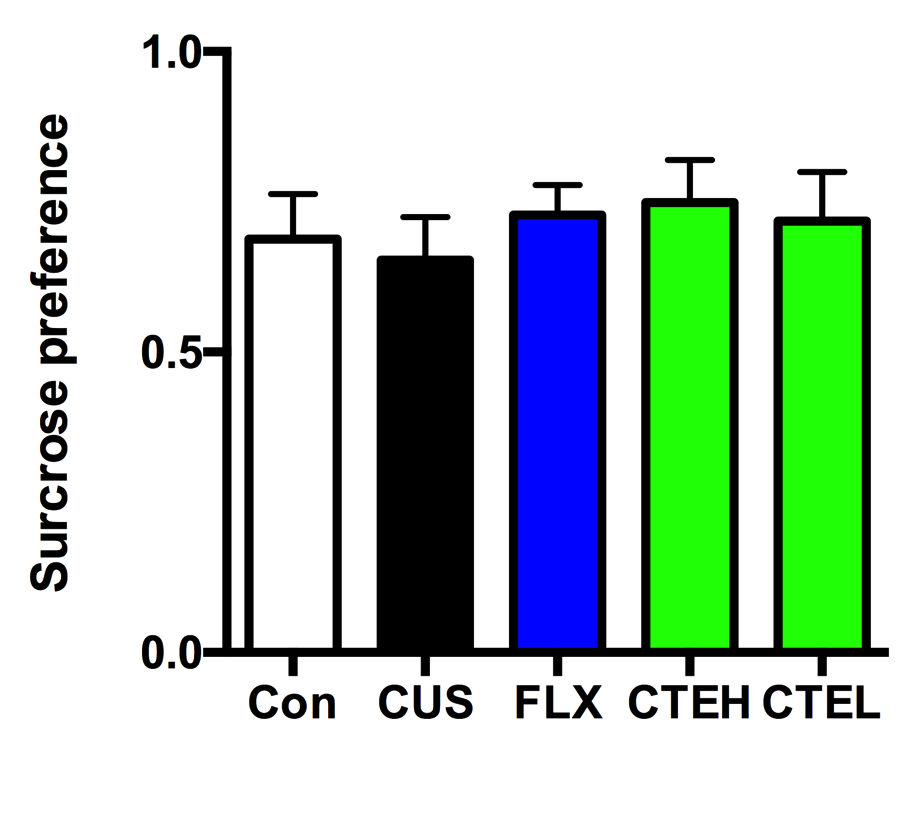


**Fig. S1** Effects of *C. tubulosa* extract of the sucrose preference test (before stress) in CUS rats. Con, control; CUS, chronic unpredictable stress; FLX, fluoxetine; CTEH: *C. tubulosa* extract high dose; CTEL: *C. tubulosa* extract low dose.


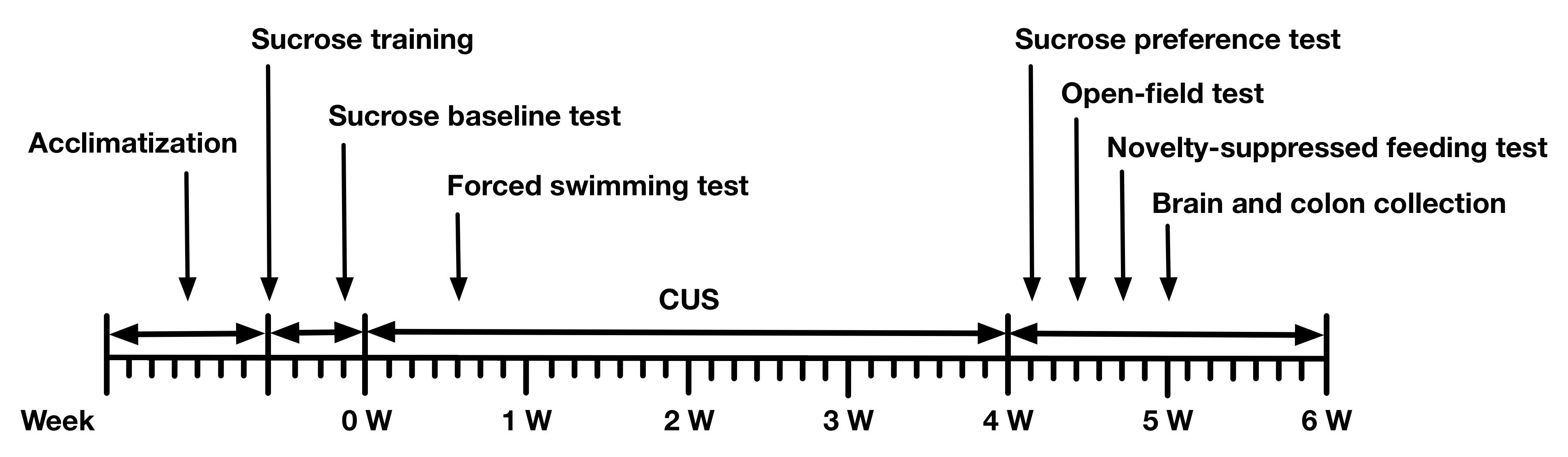


**Fig. S****2** Outline of design for chronic unpredictable stress and behavioral tests.


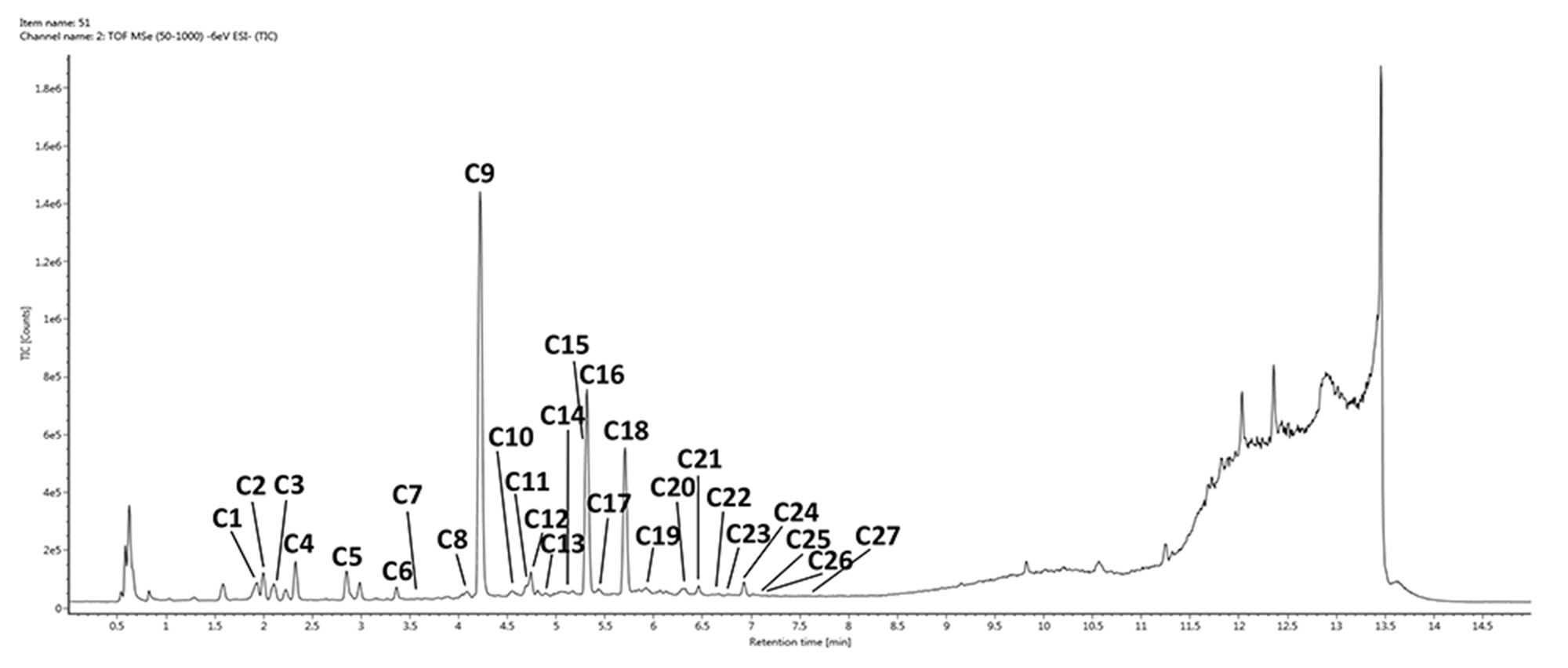


**Fig. S3** UPLC-Q-TOF-MS total ion chromatograms (TIC) of *C. tubulosa* extract.


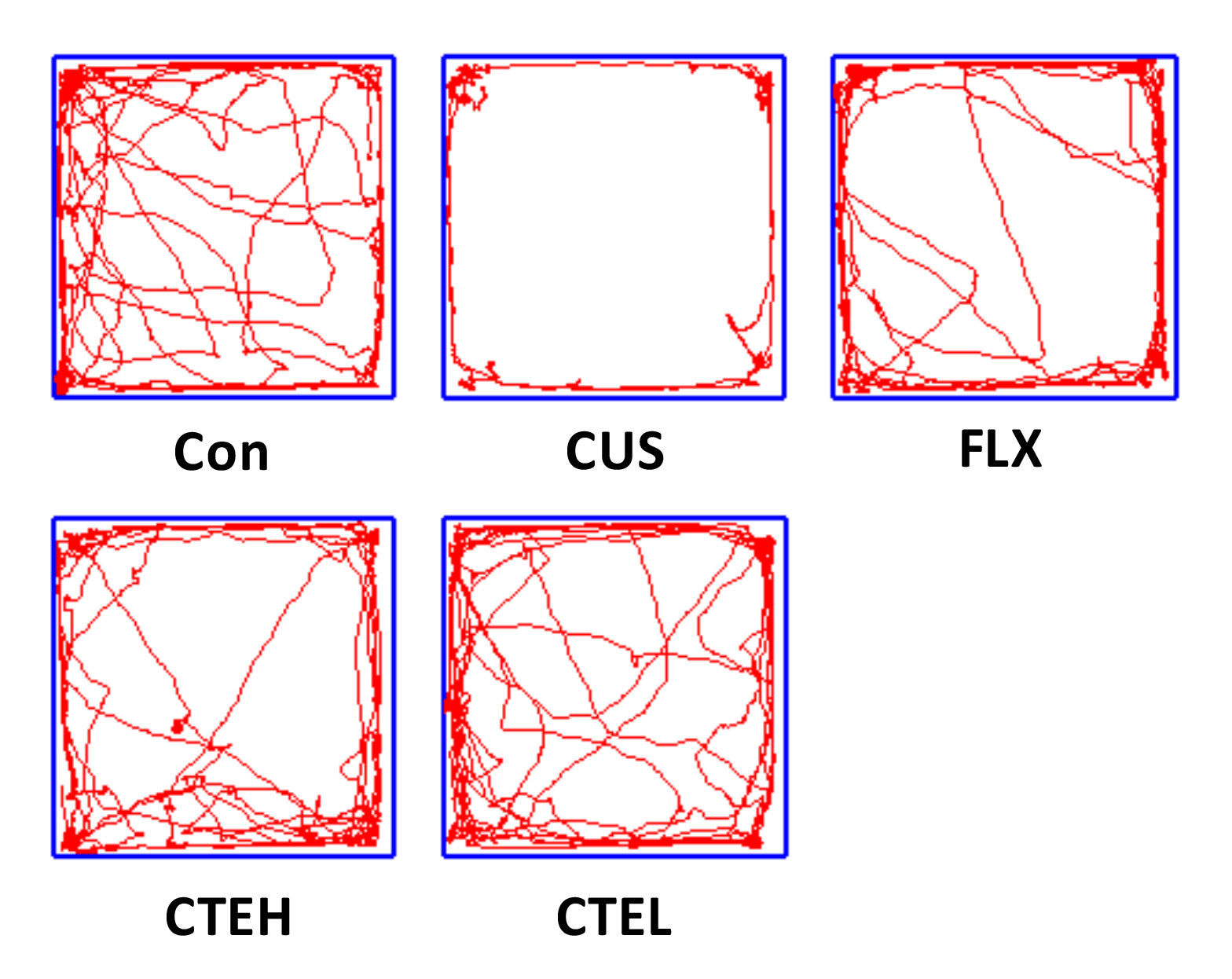


**Fig. S4** Effects of *C. tubulosa* extract of the total distances of open-field test in CUS rats. Con, control; CUS, chronic unpredictable stress; FLX, fluoxetine; CTEH: *C. tubulosa* extract high dose; CTEL: *C. tubulosa* extract low dose.


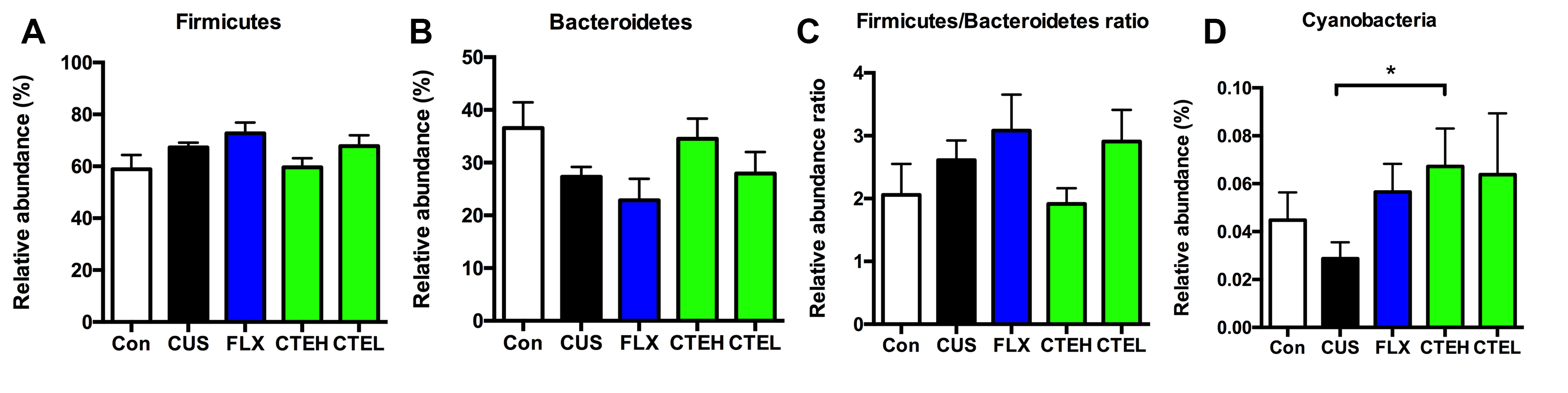


**Fig. S5** Relative abundance of selected phylum among each group. Relative abundance of Firmicutes (A), Bacteroidetes (B), Firmicutes/Bacteroidetes ratio (C), and Cyanobacteria (D). Con, control; CUS, chronic unpredictable stress; FLX, fluoxetine; CTEH: *C. tubulosa* extract high dose; CTEL: *C. tubulosa* extract low dose. **P*<0.05, (*n*=8，Mean±SEM).
